# Clinical and Radiological Characteristics of Acute Cerebrovascular Diseases Among Egyptian Patients With COVID-19 in Upper Egypt

**DOI:** 10.3389/fneur.2021.635856

**Published:** 2021-03-22

**Authors:** Eman M. Khedr, Radwa K. Soliman, Noha Abo-Elfetof, Mariam Amin, Ossama Yassin Mansour, Ahmed Aly, Ahmed F. Zaki, Mostafa Saber

**Affiliations:** ^1^Department of Neuropsychiatry, Faculty of Medicine, Assiut University Hospital, Asyut, Egypt; ^2^Department of Diagnostic and Interventional Radiology, Faculty of Medicine, Assiut University Hospital, Asyut, Egypt; ^3^Department of Public Health and Community Medicine, Faculty of Medicine, Assiut University Hospital, Asyut, Egypt; ^4^Department of Neurology Stroke and Neuro-Intervention Section, Faculty of Medicine, Alexandria University, Alexandria, Egypt; ^5^Department of Neurosurgery, Faculty of Medicine, Aswan University Hospital, Aswan, Egypt; ^6^Department of Neuropsychiatry, Faculty of Medicine, South Valley University, Qena, Egypt; ^7^Department of Neuropsychiatry, Faculty of Medicine, Aswan University Hospital, Aswan, Egypt

**Keywords:** COVID-19, cerebrovascular stroke, central nervous system, anosmia, large vessel occlusion, hemorrhagic infarction

## Abstract

**Background and Purpose:** There is little information on the acute cerebrovascular complications of coronavirus disease 2019 (COVID-19) in Egypt. The aim of this study was to estimate the proportion of acute cerebrovascular disease (CVD) among COVID-19 patients and evaluate their clinical and radiological characteristics in comparison with non-COVID-19 CVD.

**Materials and Methods:** In a retrospective study, COVID-19 patients whom presented with CVD in Assiut and Aswan University Hospitals were compared with non-COVID-19, CVD patients, admitted to Qena University Hospital, prior to the pandemic. The following data were collected: clinical history and presentation, risk factors, comorbidities, brain imaging (MRI or CT), chest CT, and some laboratory investigations.

**Results:** Fifty-five (12.5%) of the 439 patients with COVID-19 had acute CVD. Of them, 42 (9.6%) had ischemic stroke while 13 patients (2.9%) had hemorrhagic CVD. In the 250 cases of the non-COVID-19 group, 180 had ischemic stroke and 70 had hemorrhagic stroke. A large proportion of patients with COVID-19 who presented with ischemic stroke had large vessel occlusion (LVO), which was significantly higher than in non-COVID-19 patients with CVD (40 vs. 7.2%, *P* < 0.001). Comorbidities were recorded in 44 (80%) cases. In COVID-19 ischemic stroke patients, risk factors [hypertension and ischemic heart disease (IHD)] and comorbidities (hepatic and renal) were significantly higher than those in non–COVID-19 patients. In addition, 23.5% had hemorrhagic CVD, and six patients with LVO developed hemorrhagic transformation.

**Conclusion:** Acute CVD among patients with COVID-19 was common in our study. LVO was the commonest. Hypertension, IHD, and anemia are the most common risk factors and could contribute to the worsening of clinical presentation. Comorbidities were common among patients with CVD, although a large number had elevated liver enzymes and creatinine that were partially due to COVID-19 infection itself. The current results begin to characterize the spectrum of CVD associated with COVID-19 in patients in Upper Egypt.

**Registration ID:** The ID number of this study is IRB no: 17300470.

## Introduction

In most patients, severe acute respiratory syndrome coronavirus 2 (SARS-CoV-2) infection presents with a flu-like illness; neurological symptoms are most usually seen in patients with other comorbidities. Recently, some studies (1, 2) and case reports (3–6) have reported a small number of COVID-19 patients with concurrent stroke, the majority of whom had ischemic rather than hemorrhagic strokes. The World Health Organization (WHO) reported that the risk of ischemic stroke associated with COVID-19 is around 5% (7). There have also been a few cases, in patients with mild symptoms, of macrothrombosis in the internal carotid artery (8, 9). Most of the strokes occurred in young adults without cardiovascular risk factors. For example, Gunasekaran et al. (10) described a case of cerebrovascular stroke in a COVID-19 patient younger than 50 years with few risk factors for stroke. At the present time, there is little knowledge about the clinical and radiological criteria of acute cerebrovascular complications of COVID-19 in Egypt.

This retrospective study analyzes data from COVID-19 patients with acute cerebrovascular disease (CVD) who were admitted into the two largest university hospitals in Upper Egypt. We estimate the proportion of acute CVD among COVID-19 patients and evaluate their clinical and radiological characteristics in comparison to a group of patients with acute CVD without COVID-19 who had been observed 3 years prior to the pandemic in Qena University Hospital (Upper Egypt).

## Materials and Methods

Patients with suspected COVID-19 were admitted from June 1 to August 10, 2020, to two university hospitals in Upper Egypt (Assiut and Aswan), which served as quarantine areas (11). Then, all patients with COVID-19 infection, whom presented with acute CVD, were transferred to the intensive care unit (ICU) of the Neurology, Neurosurgery Hospital of Assiut and the ICU in Aswan University hospital. We used the World Health Organization (WHO) definition of stroke as “rapidly developed clinical signs of focal (or global) disturbance of cerebral function, lasting more than 24 h or leading to death, with no apparent cause other than of vascular origin” (12). We documented the latter using either computed tomography (CT) or magnetic resonance imaging (MRI) with additional data regarding demographic data, risk factors, and comorbidities. Clinical assessment was made using the National Institutes of Health Stroke Scale (NIHSS), Glasgow Coma Scale (GCS), and chest CT. Laboratory investigations included blood picture and gases, renal and liver function, and coagulation profile [prothrombin time and concentration and international normalized ratio (INR)]. D-dimer and ferritin levels were measured for a few patients when available.

Our control group was taken from retrospective data of all patients diagnosed with acute CVD within 72 h of onset and admitted to Qena University Hospital from October 1, 2015, to the end of March 2016. Qena is a Nile Valley governorate and one of the largest cities in Upper Egypt, lying midway between Assiut and Aswan governorates where they share a common culture and climate.

CT scanners included GE Bright Speed Elite 16 slice, Siemens-Somatom go UP32 slice, and Toshiba Aquilion PRIME, while MRI scanners included Philips Achieva, 1.5 T, Siemens Avanto 1.5 T, and Toshiba Ventage 1.5 T.

Only cases with imaging-confirmed stroke were included in the study. For ischemic insult, vascular territories were identified (whether venous or arterial/large or small vessel occlusion), while hemorrhagic insults were classified into intra-parenchymal (lobar, deep, and infra-tentorial) and extra-parenchymal hemorrhage (intraventricular and subarachnoid), as well as mixed intra-parenchymal and extra-parenchymal hemorrhage. A chest CT was also obtained.

The raw data supporting the findings of this study are available upon request from the corresponding authors.

### Consent and Ethics

Each patient or relative gave a written informed consent. Approval of the study was obtained from the local ethics committee of Assiut University Hospital.

### Infection of COVID-19 Was Defined as

**1-Definite COVID-19** if patients came with clinical symptoms of infection and had a positive reverse transcription polymerase chain reaction (PCR) test of respiratory samples (e.g., nasopharyngeal swab).
**2-Suspected COVID-19** if the usual clinical symptoms of COVID-19 infection but PCR were not available. Diagnosis was based on chest CT and one of the following laboratory data was positive: lymphopenia and/or ferritin level, D-dimer.

### Statistical Analysis

Data were analyzed using the Statistical Package for the Social Sciences (SPSS 25). Number and percent or means ± standard deviation (SD) were used to represent data. Demographic, risk factors, comorbidities, and other variables were compared between COVID-19 CVD patients versus non-CVD patients and also between COVID-19 CVD patients and non-COVID-19 CVD patients with the χ^2^ test. The level of significance was set at *P* < 0.05.

## Results

**Figure 1:**
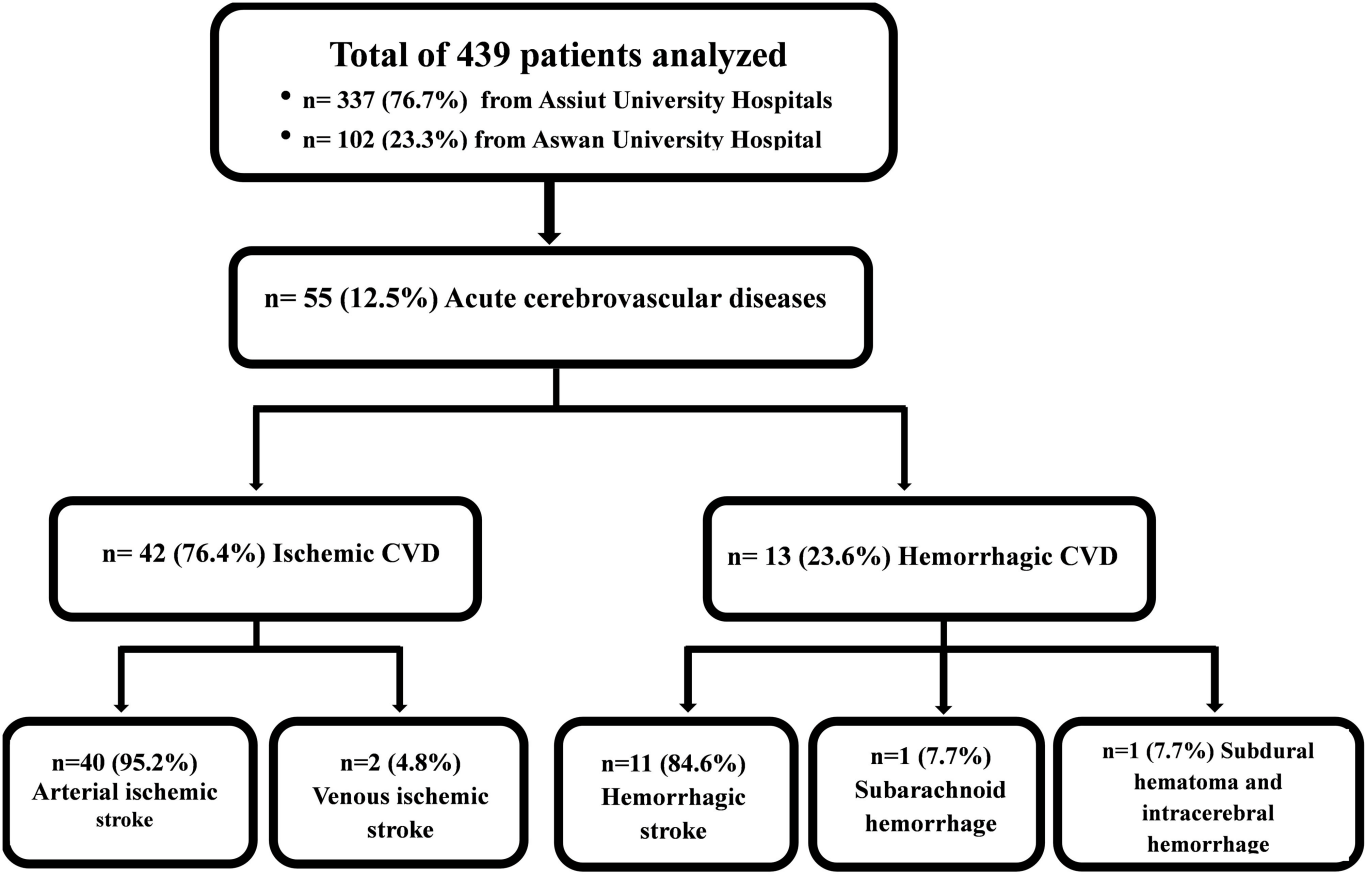
Flowchart.

Out of 439 patients with confirmed/suspected COVID-19, acute CVD was recorded in 55 cases (12.5%). Cases were classified using neuroimaging data. Here, 42 (9.6%) patients were diagnosed with ischemic arterial/venous stroke (40/2); 11 (2.5%) cases were diagnosed with hemorrhagic stroke; one case (0.2%) had combined subdural hematoma and intracerebral hematoma; and the remaining case (0.2%) had subarachnoid hemorrhage (see Figure 1 flowchart).

The mean age of the patients with COVID-19–CVD was 62.8 ± 14.1 years (range 35–90 years), with 30 (54.5%) males and 25 (45.5%) females. Twelve patients (21.8%) had a positive PCR and were diagnosed as definite COVID-19; the remaining 43 cases (78.2%) were diagnosed as suspected COVID-19 because they had fever and pulmonary symptoms plus chest CT findings of bilateral ground-glass opacities with consolidation (GGO) in addition to lymphopenia and/or elevated ferritin level or D-dimer.

Fifty-one (92.7%) out of 55 COVID-19 patients had bilateral ground-glass appearance with consolidation in CT chest. Among the constitutional symptoms of COVID-19 in the CVD group, we found that fever (89.1%) and respiratory symptoms (81.9%) were the most common manifestations, followed by headache (30.9%) and gastrointestinal tract (GIT) (21.8%) symptoms. Fatigue and malaise (9.3%) as well as dizziness and vertigo (3.6%) were least frequent. In the CVD group with COVID-19, 44 (80%) had risk factors and/or comorbidities. Comparison of demographic, clinical data, risk factors, and comorbidities between COVID-19 patients with CVD (55 cases) vs. without CVD (384 cases) showed a significantly higher mean age of COVID-19 patients with CVD than those without CVD. The percentages of fever, headache, and disturbed consciousness were significantly higher in COVID-19 with CVD than without, while fatigue, myalgia, malaise, dizziness, and vertigo were significantly lower in COVID-19 with CVD than without. In general, risk factors and comorbidities were significantly higher in COVID-19 with CVD than without (Table 1).

**Table 1:** Demographic and clinical data of COVID patients with CVD vs. without CVD.

**Demographics**	**CVD patients** **(*n* = 55)**	**Non-CVD** **(*n* = 384)**	***P*-value**
**Age (years)**			
Mean ± SD	62.8 ± 14.1	49.5 ± 16.9	<0.001
Range	35–90	18–86	
≤ 50 n (%)	12 (21.8 %)	183 (47.7%)	<0.001
>50 n (%)	43 (78.2%)	201 (52.3%)	
**Sex** ***n*** **(%)**			
Male	30 (54.5%)	194 (50.5%)	0.577
Female	25 (45.5%)	190 (49.5%)	
**Presenting symptoms** ***n*** **(%)**			
Fever	49 (89.1%)	278 (72.4%)	0.008
Respiratory symptoms	45 (81.9%)	283 (73.7%)	0.195
Headache	17 (30.9%)	67 (17.4%)	0.018
GIT symptoms	12 (21.8%)	81 (21.1%)	0.902
Fatigue, myalgia and malaise	5 (9.1%)	170 (44.3%)	<0.001
Dizziness and vertigo	2 (3.6%)	61 (15.9%)	0.015
Disturbed consciousness	27 (49.1%)	17 (4.4%)	<0.001
**Comorbid risk factor and comorbidities** ***n*** **(%)**			
Hypertension	32 (58.2%)	140 (36.5%)	0.002
Ischemic heart disease	14 (25.4%)	42 (10.9%)	0.003
Rheumatic heart disease	2 (3.6%)	1 (0.26%)	0.005
Atrial fibrillation	1 (1.8%)	2 (0.52%)	0.275
Diabetes mellitus	17 (30.9%)	130 (33.9%)	0.665
Liver disease	5 (9.1%)	10 (2.6%)	0.013
Renal disease	8 (14.5%)	16 (4.2%)	0.001
Chronic pulmonary disease	1 (1.8%)	29 (7.6%)	0.115
No risk factor or comorbidities	11 (20.0%)	159 (41.4%)	0.002

Table 2 compares demographics, clinical, and risk factors and comorbidities of COVID-19 ischemic stroke patients (42 patients) and non-COVID-19 ischemic stroke patients (180 patients). The mean age of COVID-19 patients was significantly higher than that of non-COVID-19 patients, while no significant difference was found in terms of sex. Hypertension and ischemic heart disease (IHD) were significantly higher in COVID-19 than those in non-COVID-19 patients as risk factors for stroke. Also, comorbidities (hepatic and renal disease) were significantly higher in COVID-19 than non-COVID-19. NIHSS and GCS were significantly worse in COVID-19 compared with non-COVID-19 ischemic stroke patients with a higher percentage of patients presenting with a disturbed level of consciousness. Table 3 shows the comparison between hemorrhagic stroke in COVID-19 and non-COVID-19 patients. There were no significant differences between groups in demographics, risk factors, and comorbidities, or in clinical presentation. However, there were significantly higher National Institutes of Health Stroke Scale/Score (NIHSS) and lower Glasgow Coma Scale (GCS) scores in the COVID-19 group than the non-COVID-19 group, with a higher percentage of disturbed consciousness in the COVID-19 group.

**Table 2:** Comparison between Covid-19 and non-covid-19 ischemic stroke patients in demographic, risk factors, comorbidities, and clinical presentation.

**Demographic, risk factors clinical presentation and comorbidities**	**COVID-19 ischemic stroke** **(*n* = 42)**	**Non-COVID-19 ischemic stroke** **(*n* = 180)**	*P*-value^*^
**Age (years)**			
Mean ± SD	64.8 ± 13.7	56.1 ± 1.5	<0.001
Range	37–90	33–85	
Age ≤ 50 n (%)	8 (19.0%)	56 (31.1%)	0.120
Age >50 n (%)	34 (81.0%)	124 (68.9%)	
**Sex** ***n*** **(%)**			
Male	22 (52.4%)	84 (46.7%)	0.562
Female	20 (47.6%)	96 (53.3%)	
**Stroke risk factors and comorbidities** ***n*** **(%)**			
Hypertension	23 (54.7%)	52 (28.8%)	0.001
Ischemic heart disease	13 (30.9%)	15 (8.3%)	0.001
Rheumatic heart disease	2 (4.7%)	6 (3.3%)	0.654
Diabetes mellitus	14 (33.3%)	53 (29.4%)	0.621
Atrial fibrillation	2 (4.7%)	19 (10.6%)	0.247
Hepatic disease	3 (7.1%)	2 (1.1%)	0.017
Renal diseases	8 (19%)	5 (2.8%)	0.001
No risk factor or comorbidities	10 (23.8%)	35(19.4%)	0.526
**Clinical presentation**			
NIHSS Mean ± SD (range)	13.8 ± 5.6 (4–24)	9.2 ± 5.4	<0.001
GCS Mean ± SD (range)	9.5 ± 4.5 (0–15)	13.3 ± 1.9	<0.001
DCL n (%)	15 (35.7%)	3 (1.7%)	<0.001

*National Institutes of Health Stroke Scale, GCL, Glasgow Coma Scale; DCL, Disturbed Conscious Level*.

* *Students' T-test and Chi-square test were used*.

**Table 3:** Difference between Covid-19 and non-covid-19 hemorrhagic stroke patients in demographic, risk factors and comorbidities.

**Demographic, Stroke risk factors and comorbidities and clinical presentation**	**COVID-19 hemorrhagic CVD (*n* = 13)**	**Non-COVID-19 hemorrhagic CVD** **(*n* = 70)**	***P*-value**
**Age (years)**			
Mean ± SD	57.4 ± 13.5	50.4 ± 13.3	0.204
Range	35–80	19–99	
Age ≤ 50 n (%)	5 (38.5%)	38 (54.3%)	0.294
Age >50 n (%)	8 (61.5%)	32 (45.7%)	
**Sex** ***n*** **(%)**			
Male	8 (61.5%)	32 (45.7%)	0.294
Female	5 (38.5%)	38 (54.3%)	
**Stroke risk factors and comorbidities** ***n*** **(%)**			
Hypertension	8 (61.5%)	46 (65.7%)	0.771
Ischemic heart disease	1 (7.7%)	1 (4.2%)	0.176
DM	3 (23.1%)	23 (2.9%)	0.485
Atrial fibrillation	1 (7.7%)	1 (1.4%)	0.176
Chronic pulmonary disease	1 (7.7%)	1 (1.4%)	0.176
Hepatic disease	2 (15.4%)	4 (5.7%)	0.216
Renal disease	0	1(1.4%)	–
No risk factor or comorbidities	1 (7.7%)	5 (7.1%)	0.891
**Clinical presentation**			
NIHSS Mean ± SD (range)	16.1 ± 3.2 (9–22)	10.6 ± 6.2	<0.001
GCS Mean ± SD (range)	8.7 ± 3.4 (5–159)	12.3 ± 2.6	<0.001
DCL n (%)	6 (46.1%)	2 (2.8%)	<0.001

*DM, Diabetes Mellites; HCV, Hepatitis C Virus; RHD, Rheumatic Heart Disease; NIHSS, National Institutes of Health Stroke Scale; GCL, Glasco Coma Scale; DCL, Disturbed Conscious Level*.

### Laboratory Data

Regarding the blood picture: 24 (43.6%) cases had leukocytosis (19 ischemic and five hemorrhagic), 26 (47.3%) cases had lymphopenia (19 in ischemic and seven in hemorrhagic stroke), and 26 patients (47.3%) had microcytic hypochromic anemia (23 ischemic and three hemorrhagic). Eight (15.5%) had prolonged prothrombin time (PTT), and five (9.1%) had decreased prothrombin concentration (five in ischemic stroke and three in hemorrhagic).

Interestingly, in ischemic stroke (42 cases), only three patients had a history of liver disease among COVID-19 patients. However, during admission, 16 (38.1%) cases had elevated liver enzymes of whom three had increased PTT and decreased prothrombin concentration. Eight patients (19%) had a history of kidney diseases. Yet, 16 cases (38.1%) had elevated blood urea and creatinine (renal impairment) during admission.

In hemorrhagic stroke (13 cases), only two patients had a history of liver disease. However, during admission, four (36.4%) cases had elevated liver enzymes of whom three had increased PTT and decreased prothrombin concentration. There were no patients with a history of kidney disease. Yet, during admission, four cases had elevated blood urea and creatinine (renal impairment). In total, six patients had both impaired renal and elevated liver enzymes.

### Neuroimaging

Based on radiological findings, there were no significant differences between COVID-19 and non-COVID-19 patients in terms of the incidence of either ischemic or hemorrhagic CVD (details are provided in Table 4). However, COVID-19 patients had a significantly higher rate of large vessel occlusion compared to non-COVID-19 patients [40% in COVID-19 patients vs. 7.2% in non-COVID-19 patients (*P* < 0.001)]. Furthermore, there was a significantly higher rate of hemorrhagic transformation (of arterial ischemic stroke) in COVID-19 patients (14.3%) compared to non-COVID-19 patients (1.6%), with *P* < 0.001.

**Table 4:** Radiological findings of 55 COVID-19 patients with cerebrovascular diseases (CVD).

**Radiological findings**	**COVID-19 CVD** **(*n* = 55)**	**Non-COVID-19 CVD** **(*n* = 250)**	***P*-value**
I-^*^Arterial/venous ischemic stroke	**42 (76.4%)**	**180 (72%)**	**0.510**
1-Anterior circulation	31 (56.4%)	125 (50%)	0.393
A-Large artery occlusion	22 (40.0%)	18 (7.2%)	<0.001
B-Small vessels occlusion (Territories of MCA)	9 (16.4%)	107 (42.85)	<0.001
2-Posterior circulation	9 (16.4%)	47 (29.6%)	0.673
A-large vessel occlusion	2 (3.6%)	2 (0.8%)	0.094
B-Small vessels occlusion	7 (12.7%)	45 (18%)	0.347
3-Mixed anterior and posterior circulation	0	6 (2.4%)	–
4-Venous stroke:	2 (3.6%)	2 (0.8%)	0.094
II-Hemorrhagic CVD	13 (23.6%)	70 (28%)	0.510
1-Intra-parenchymal	6 (10.9%)	49 (19.6%)	0.129
Deep	2 (3.6%)	28 (11.2%)	0.088
Lobar	2 (3.6%)	12 (4.8%)	0.998
Lobar and deep	0	2 (0.8%)	–
Infra-tentorial	2 (3.6%)	7 (2.8%)	0.740
2-Extra-parenchymal	3 (5.5%)	6 (2.4%)	0.226
Intra-ventricular	2 (3.6%)	3 (1.2%)	0.198
Subarachnoid	1 (1.8%)	3 (1.2%)	0.715
3-Mixed intra and extra- parenchymal	4 (7.2%)	15 (6%)	0.724

* *Hemorrhagic transformation was observed in 6 cases, one internal carotid artery, 3 out of 13 right middle cerebral artery occlusion (MCAO), and 2 left middle cerebral artery occlusion (MCAO), while only 3 cases out of 180 non-COVID ischemic stroke had Hemorrhagic transformation with significant difference (P < 0.001). 4 young adult had large vessel occlusion*.

*CVD, cerebrovascular diseases; MCA, middle cerebral artery*.

**Figure 2:**
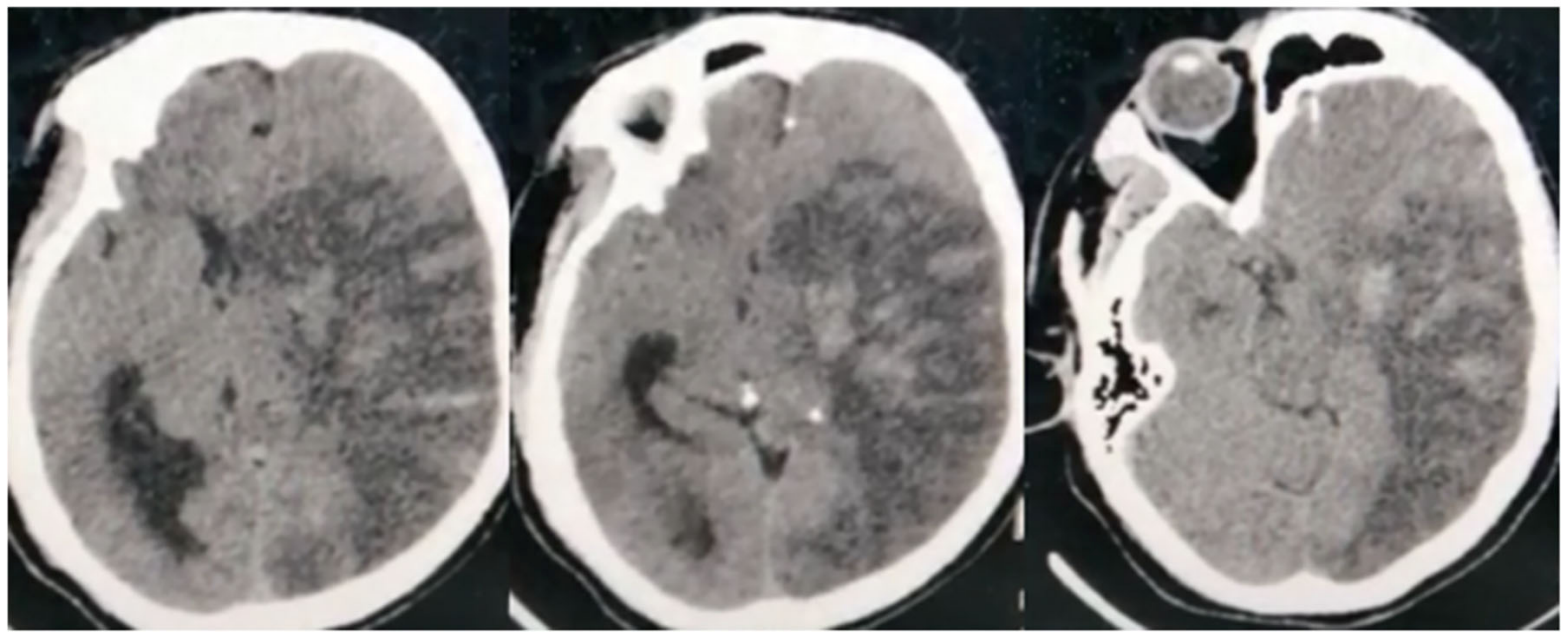
Non-contrast CT brain of a 45-year-old male shows subacute left middle cerebral artery (MCA) territory infarct (large vessel occlusion), associated with marked edema excreting mass effect on the lateral ventricle, and midline shift. Noted multiple hyperdense patches within the infarct representing hemorrhagic transformation.

**Figure 3:**
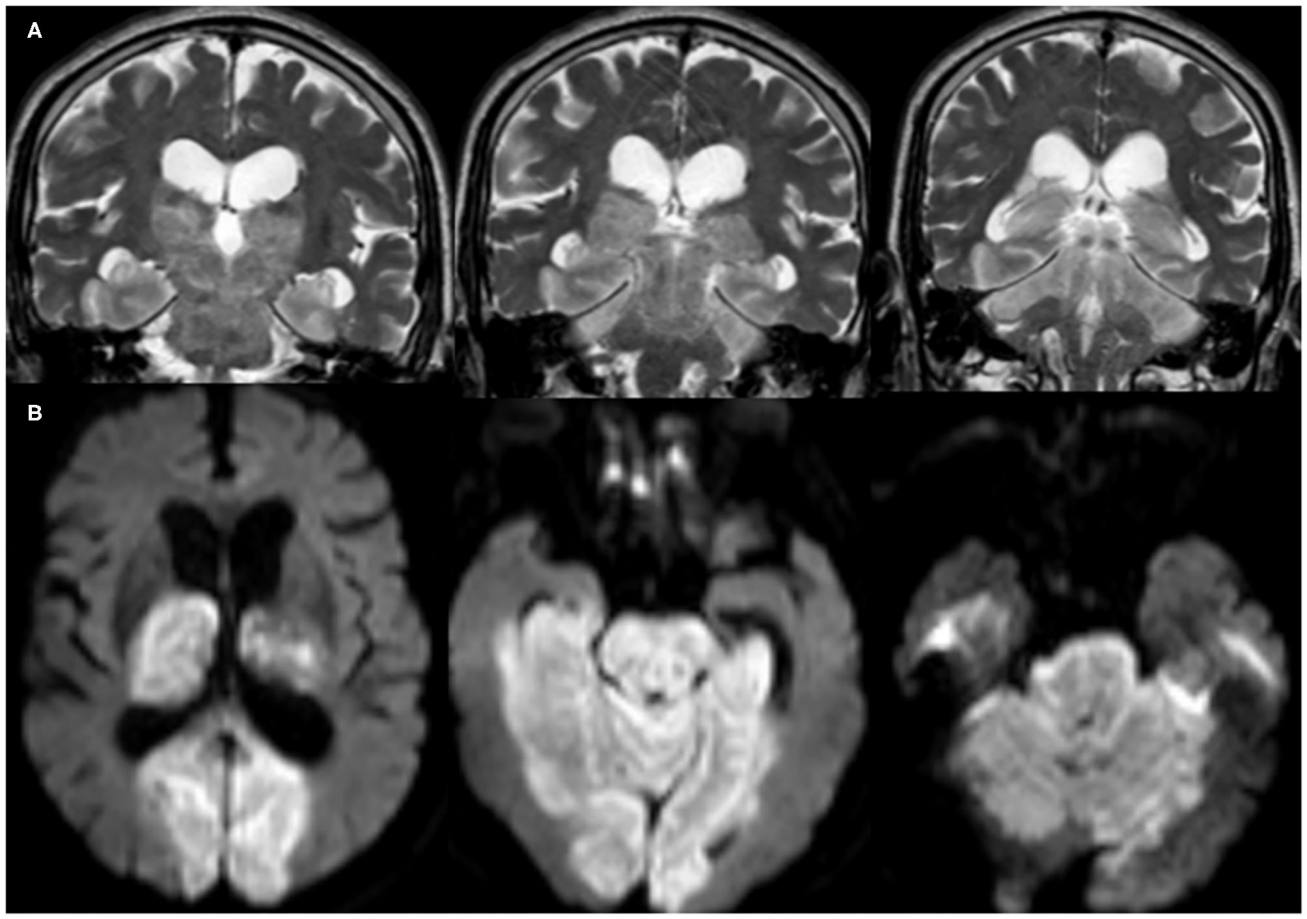
Brain MRI, **(A)** coronal T2-weighted imaging (T2WI), and **(B)** axial diffusion-weighted imaging (DWI) of a 76-year-old male patient show acute infarct (large vessel occlusion) involving the basilar artery territories (brain stem, bilateral thalami, occipital and inferior temporal lobes, as well as the cerebellum).

In total, 42 (76.4%) COVID-19 cases had ischemic CVD, which was arterial in 40 (72.7%) cases and cerebral sinus venous thrombosis (CSVT) in two cases (3.6%). The anterior circulation was affected in 31 (56.4%) cases, while 22 (40%) had large vessel occlusion. Out of the latter, eight (14.5%) patients had occlusion of the left middle cerebral artery (MCA), 13 (23.6%) had right MCA occlusion, and one patient (1.8%) had occlusion of the left internal carotid artery. Hemorrhagic transformation developed in six (10.9%) patients (Figure 2). Large vessel occlusion occurred in four young patients. Small vessel occlusion (MCA territories) was observed in nine (16.4%) cases. The posterior circulation was affected in nine (16.4%) patients, of whom two (3.6%) had LVO (basilar artery) (Figure 3).

**Figure 4:**
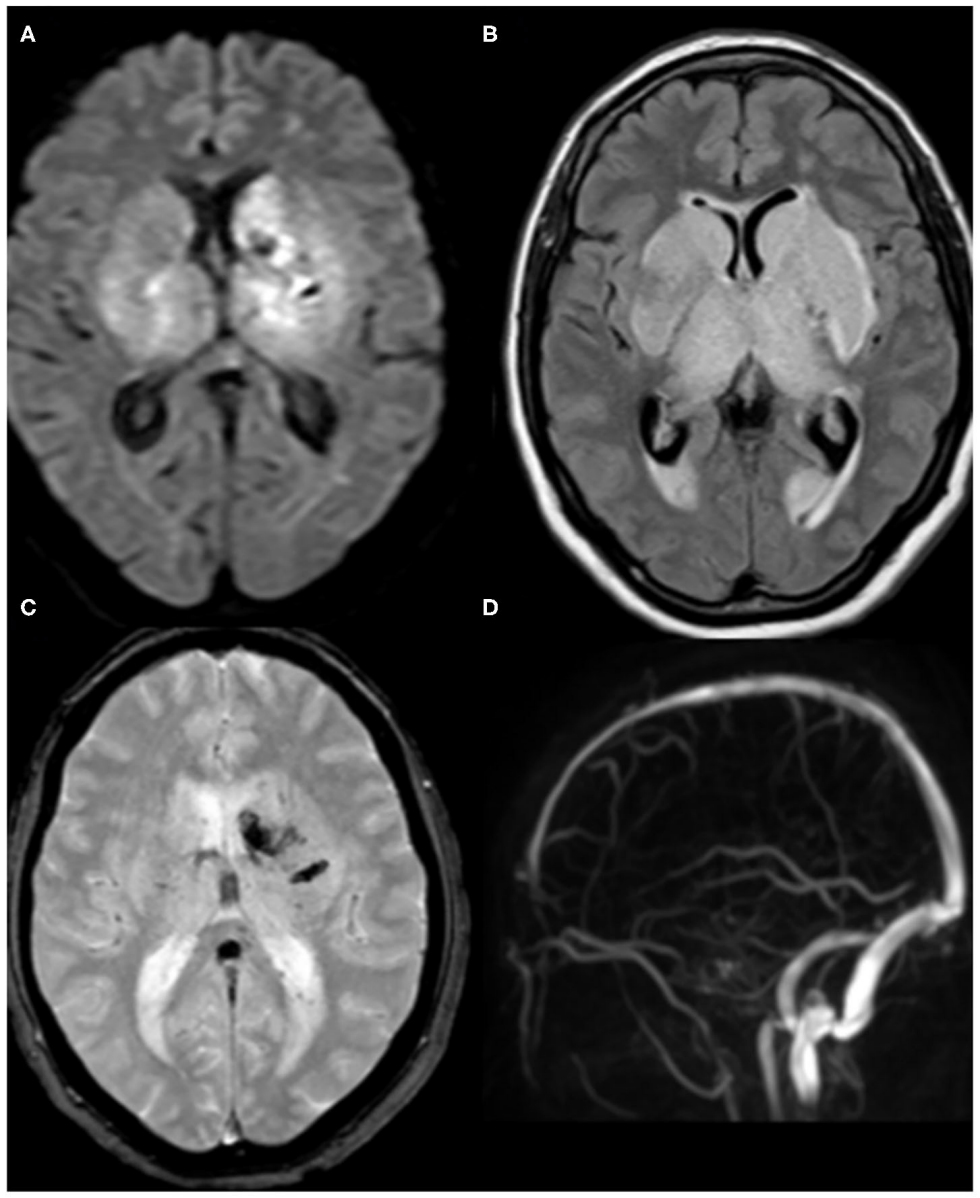
Brain MRI of a 62-year-old female patient. **(A)** axial DWI and **(B)** Fluid-attenuated inversion recovery (FLAIR) demonstrate acute infarction involving bilateral thalami and basal ganglia. **(C)** T2^*^-weighted imaging (T2^*^WI) shows associated hemorrhagic foci at the left side. **(D)** The corresponding magnetic resonance venography (MRV) reveals the absence of the normal flow in the deep cerebral veins. Findings are consistent with venous infarction secondary to deep cerebral venous thrombosis.

With regard to venous stroke, one patient had a deep cerebral vein thrombosis with bilateral thalamic and basal ganglia infarction (Figure 4), and the other patient had left transverse and sigmoid sinus thrombosis with parenchymal infarction.

**Figure 5:**
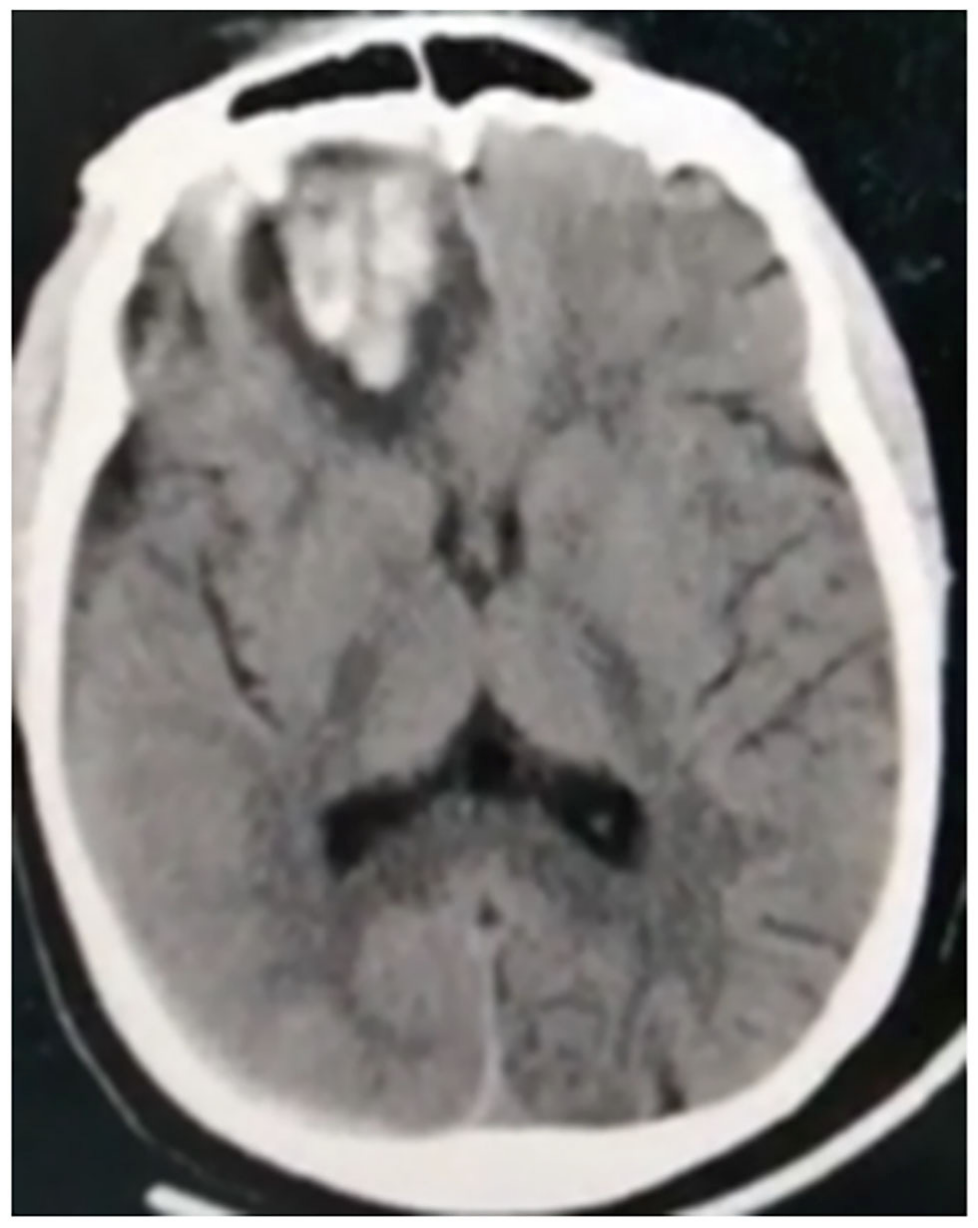
Non-contrast CT of a 38-year-old male patient shows intra-parenchymal right inferior frontal hematoma.

Hemorrhagic CVD was observed in 13 (23.6%) cases. Intra-parenchymal hemorrhage (Figure 5) occurred in six (10.9%) patients, two of which (3.6%) had deep (basal ganglionic) hemorrhage, two (3.6%) had lobar (frontotemporal and left inferior frontal lobe) hemorrhage, and two (3.6%) had infratentorial (pontine) hemorrhage. Extra-parenchymal hemorrhage occurred in three (5.5%) cases in which there was no CT angiography evidence of aneurysm or arteriovenous malformation. Of these, two (3.6%) had intraventricular hemorrhage, and one (1.8%) had subarachnoid hemorrhage. In addition, mixed intraventricular and basal ganglia hemorrhage occurred in three (5.4%) cases, while one patient (1.8%) had mixed subdural and inter-parenchymal hematoma, with no history of trauma (details illustrated in Table 4).

## Discussion

The main finding of this study is the high frequency of CVD in comparison to previous studies (13, 14): 55 out of 439 COVID-19 patients had CVD (12.5%). However, this is likely to be an overestimate of general prevalence, since patients with mild symptoms were asked to isolate at home, and only patients with moderate to severe symptoms or those with complications or comorbidities were admitted. Most of the strokes were ischemic, but hemorrhagic strokes and cerebral sinus venous thrombosis (CSVT) were observed.

Previous papers generally reported a lower frequency of CVD. In a Chinese cohort of 214 confirmed COVID-19 patients, CVD was seen in six patients (2.8%) (2). Requena et al. (15) reported 21 (1.02%) cases with an acute ischemic stroke and four (0.2%) with an intracranial hemorrhage in a sample of 2,050 patients with confirmed SARS-CoV-2. In a systematic review of 80 COVID-19 articles, Fraiman et al. (16) found a total of 226 cases of ischemic stroke, 35 cases of intracranial bleeding, and 14 cases of venous sinus thrombosis. The same distribution was observed in the current study: 42 cases had ischemic stroke (9.6% of 439) and 13 patients (2.5% of 439) had hemorrhagic CVD. Li et al. (13) reported only 13 cases (5.9%) of CVD out of 221 COVID-19 patients; 11 (84.6%) were diagnosed as ischemic stroke, one (7.7%) had cerebral hemorrhage, and the other (7.7%) had CSVT. In the case series of Reddy et al. (14) (12 cases) 10 patients had an ischemic stroke, of whom one suffered from hemorrhagic transformation and only two had intracerebral hemorrhage. Few other studies have reported cerebrovascular complications in COVID-19 (1, 2). A small number of case series have also described patients with COVID-19 and concurrent stroke (3, 5).

### Four Interesting Findings in the Current Study

First, our COVID-19 patients with ischemic stroke had a significantly higher mean age than non-COVID-19 patients. The same result was observed by Katz et al. (17), who reported that 68 COVID-19-positive stroke patients were older than 449 non-COVID-19 stroke patients. In contrast, Wang et al. (18) found that the mean age of patients in several thrombectomy case series of COVID-19 in New York City was 52.8 years. Another large medical center in New York reported that patients with COVID-19 who presented with stroke were younger than a control group of patients with stroke without SARS-CoV-2 infection (19). In contrast, Fraiman et al. (16) in their systematic review found that the mean age of COVID-19 patients with ischemic stroke was 64.16 ± 14.73 years, similar to the present data.

Second, the frequency of patients with a positive history of hypertension and IHD as well as hepatic and renal disease was significantly higher in COVID-19 than non-COVID-19 ischemic stroke patients. In total, 76.2% of COVID-19 ischemic stroke patients had preexisting risk factors. Consistent with our findings, Tiwari et al. (20) also reported that 81% of COVID-19 patients presenting with ischemic stroke had previous known vascular risk factors. COVID-19 cases are also more commonly associated with diabetes mellitus (DM), arterial hypertension (AH), and atrial fibrillation (AF) (16).

The occurrence of ischemic stroke in patients with COVID-19 may be due to competitive blockage of the angiotensin-converting enzyme 2 (ACE2) by the SARS-CoV-2 virus (21). This downregulates ACE2 expression, leading to fluctuations in blood pressure and an increase in the possibility of cerebrovascular accidents. This explanation is compatible with the significantly higher number of patients with COVID-19 who presented with hypertension (54.7%) in comparison to non-COVID-19 patients (28.8%). In addition, preexisting IHD seems to be linked with worse clinical presentation similar to the results of Guan et al. (22) and Wang et al. (23). COVID-19 itself can also induce myocardial injury, arrhythmia, acute coronary syndrome, and venous thromboembolism (24, 25).

A large number of patients [26 or 47.3%; 23 (54.8%)] with ischemic stroke and three with hemorrhagic stroke had microcytic hypochromic anemia. Unfortunately, this was not recorded in our non-COVID-19 patients. Nevertheless, our results are consistent with those of Chen et al. (26) who found that 51% of 99 COVID-19 patients transferred to Jinyintan Hospital showed a decreasing tendency in hemoglobin levels. Another study on 1,099 laboratory-confirmed COVID-19 cases found that severe patients had significantly lower hemoglobin levels than those diagnosed as non-severe cases (22). Anemia is considered a hyperkinetic state that disturbs endothelial adhesion molecule genes that may lead to thrombus formation. Furthermore, blood flow augmentation and turbulence may result in the migration of this thrombus, thus producing artery-to-artery embolism.

An Important aspect of the present study was the number of COVID-19–stroke patients who had elevated liver enzymes and elevated blood urea and creatinine compared with the number of patients with history of no comorbidities. This confirms the assumption that COVID-19 infection can lead to multiorgan symptoms (affecting liver and kidney), which may worsen the clinical presentation (as measured by NIHSS) and lead to the higher percentage of patients presenting with disturbed consciousness and lower GCS in comparison to non-COVID-19 stroke patients. Our results are supported by a study of Dmytriw et al. (27) who reported that the mortality rate of patients with stroke who were COVID-19 positive was greater than that previously reported in acute ischemic stroke alone, suggesting an interaction that needs further investigation (28).

In the present study, 11 out of the 55 patients had no apparent risk factors for CVD and no associated comorbidities suggesting that mechanisms peculiar to COVID-19 may be responsible. These could be related to direct viral invasion and inflammation of the blood vessel walls leading to endotheliitis (6, 29), as well as induction of a “cytokine storm” as explained by Mangalmurti and Hunter in 2020 (30).

The third significant finding was the large proportion of COVID-19 patients who presented with ischemic stroke and large vessel occlusion, which was significantly higher compared with non-COVID-19 patients (40 vs. 7.2%, *P* < 0.001). These results are consistent with findings reported recently by Kihira et al. (31) who focused mainly on large vessel occlusion in COVID-19. Furthermore, Fraiman et al. (16) in their systematic review of COVID-19 stroke patients found that 105/226 (46.5%) patients had LVO. As mentioned above, an increase in the risk of vascular thrombosis and embolism is likely responsible for such a high incidence of large vessel occlusion. Overall, our analyses indicate that COVID-19 patients are more liable to serious CVD complications. Therefore, they should be monitored closely.

A final point of interest is that 23.5% of our patients had hemorrhagic CVD, and six patients with large vessel occlusion developed hemorrhagic transformation.

The pathogenesis of hemorrhagic stroke in the setting of COVID-19 may be related to the fluctuations in blood pressure as previously described and by the affinity of the SARS-CoV-2 for ACE2 receptors, which are expressed in endothelial and arterial smooth muscle cells in the brain and allow the virus to damage intracranial blood vessels and rupture the wall (32). The secondary hemorrhagic transformation of ischemic strokes observed in the present study may also relate to endothelial damage accompanying COVID-19 (33).

## Conclusion

COVID-19-associated CVD was common in our study, with LVO as the commonest type of stroke. Hypertension, IHD, and anemia were the most common risk factors and could potentially worsen clinical presentation. Comorbidities were common among patients with CVD; however, elevated liver enzymes and creatinine in a large number of cases may be partially due to COVID-19 infection itself. The current results begin to characterize the spectrum of CVD associated with COVID-19 patients in Egypt.

### Limitation of the Study

One of the main limitations of this study is the large number of patients who had not received a PCR test.

## Data Availability Statement

The raw data supporting the conclusions of this article will be made available by the authors, without undue reservation.

## Ethics Statement

The studies involving human participants were reviewed and approved by Local Ethical Committee of Assiut University Hospital. The patients/participants provided their written informed consent to participate in this study.

## Author Contributions

EK contributed to the study conception, design of the work, statistical analysis, and critical revision of the manuscript. NA-E and MS contributed to the study conception, design of the work, and drafting of the manuscript. RS contributed to the study conception, interpretation of neuroimaging, preparing the radiological figures, and drafting of the manuscript. AZ recruited data of non-COVID-19 stoke patients from Qena University Hospital. MA recruited the COVID-19 stoke cases and performed the analysis. OM and AA contributed to the drafting and critical revision of the manuscript. All authors gave final approval of the version to be published.

## Conflict of Interest

The authors declare that the research was conducted in the absence of any commercial or financial relationships that could be construed as a potential conflict of interest.

## References

[1] Huang C Wang Y Li X Ren L Zhao J Hu Y. Clinical features of patients infected with 2019 novel coronavirus in Wuhan, China. Lancet (London, England). (2020) 395:497–506. 10.1016/S0140-6736(20)30183-5 31986264 PMC7159299

[2] Mao L Jin H Wang M Hu Y Chen S He Q. Neurologic manifestations of hospitalized patients with coronavirus disease 2019 in Wuhan, China. JAMA Neurol. (2020) 77:683–90. 10.1001/jamaneurol.2020.1127 32275288 PMC7149362

[3] Avula A Nalleballe K Narula N Sapozhnikov S Dandu V Toom S. COVID-19 presenting as stroke. Brain Behav Immun. (2020) 87:115–9. 10.1016/j.bbi.2020.04.077 32360439 PMC7187846

[4] Beyrouti R Adams ME Benjamin L Cohen H Farmer SF Goh YY. Characteristics of ischaemic stroke associated with COVID-19. J Neurol Neurosurg Psychiatry. (2020) 91:889–91. 10.1136/jnnp-2020-323586 32354768 PMC7231545

[5] Oxley TJ Mocco J Majidi S Kellner CP. Large-vessel stroke as a presenting feature of Covid-19 in the young. N Engl J Med. (2020) 382:e60. 10.1056/NEJMc2009787 32343504 PMC7207073

[6] Zhang Y Xiao M Zhang S Xia P Cao W Jiang W. Coagulopathy and antiphospholipid antibodies in patients with Covid-19. N Engl J Med. (2020) 382:e38. 10.1056/NEJMc2007575 32268022 PMC7161262

[7] Qureshi AI Abd-Allah F Al-Senani F Aytac E Borhani-Haghighi A Ciccone A. Management of acute ischemic stroke in patients with COVID-19 infection: report of an international panel. Int J Stroke. (2020) 15:540–54. 10.1177/1747493020923234 32362244

[8] Fara MG Stein LK Skliut M Morgello S Fifi JT Dhamoon MS. Macrothrombosis and stroke in patients with mild Covid-19 infection. J Thromb Haemost. (2020) 18:2031–3. 10.1111/jth.14938 32464707 PMC7283879

[9] Mohamud AY Griffith B. Intraluminal carotid artery thrombus in COVID-19: another danger of cytokine storm? AJNR Am J Neuroradiol. (2020) 41:1677–82. 10.3174/ajnr.A6674 32616585 PMC7583117

[10] Gunasekaran K Amoah K Rajasurya V Buscher MG. Stroke in a young COVID-19 patient. QJM. (2020) 113:573–4. 10.1093/qjmed/hcaa177 32442268 PMC7313834

[11] Khedr EM Abo-Elfetoh N Deaf E Hassan HM Amin MT Soliman RK. Surveillance study of acute neurological manifestations among 439 Egyptian patients with COVID-19 in Assiut and Aswan University Hospitals. Neuroepidemiology. (2021) 1–10. 10.1159/000513647 [Epub ahead of print]. 33631765 PMC8018217

[12] Aho K Harmsen P Hatano S Marquardsen J Smirnov VE Strasser T. Cerebrovascular disease in the community: results of a WHO collaborative study. Bull World Health Organ. (1980) 58:113–30. 6966542 PMC2395897

[13] Li Y Wang M Zhou Y. Acute cerebrovascular disease following COVID-19: a single center, retrospective, observational study (3/3/2020). Stroke Vasc Neurol. (2020) 5:279–84. 10.2139/ssrn.3550025 32616524 PMC7371480

[14] Reddy ST Garg T Shah C Nascimento FA Imran R Kan P. Cerebrovascular disease in patients with COVID-19: a review of the literature and case series. Case Rep Neurol. (2020) 12:199–209. 10.1159/000508958 32647526 PMC7325208

[15] Requena M Olivé-Gadea M Muchada M García-Tornel Á Deck M Juega J. COVID-19 and stroke: incidence and etiological description in a high-volume center. J Stroke Cerebrovasc Dis. (2020) 29:105225. 10.1016/j.jstrokecerebrovasdis.2020.105225 33066917 PMC7405833

[16] Fraiman P Godeiro Junior C Moro E Cavallieri F Zedde M. COVID-19 and cerebrovascular diseases: a systematic review and perspectives for stroke management. Front Neurol. (2020) 11:574694. 10.3389/fneur.2020.574694 33250845 PMC7674955

[17] Katz JM Libman RB Wang JJ Sanelli P Filippi CG Gribko M. Cerebrovascular complications of COVID-19. Stroke. (2020) 51:e227–e31. 10.1161/STROKEAHA.120.031265 32757751 PMC7467046

[18] Wang A Mandigo GK Yim PD Meyers PM Lavine SD. Stroke and mechanical thrombectomy in patients with COVID-19: technical observations and patient characteristics. J Neurointerv Surg. (2020) 12:648–53. 10.1136/neurintsurg-2020-016220 32451359

[19] Yaghi S Ishida K Torres J Mac Grory B Raz E Humbert K. SARS-CoV-2 and Stroke in a New York Healthcare System. Stroke. (2020) 51:2002–11. 10.1161/STROKEAHA.120.030335 32432996 PMC7258764

[20] Tiwari A Berekashvili K Vulkanov V Agarwal S Khaneja A Turkel-Parella D. Etiologic subtypes of ischemic stroke in SARS-CoV-2 patients in a cohort of New York city hospitals. Front Neurol. (2020) 11:1004. 10.3389/fneur.2020.01004 33041972 PMC7527497

[21] Zhu H Rhee JW Cheng P Waliany S Chang A Witteles RM. Cardiovascular complications in patients with COVID-19: consequences of viral toxicities and host immune response. Curr Cardiol Rep. (2020) 22:32. 10.1007/s11886-020-01302-4 32318865 PMC7171437

[22] Guan WJ Ni ZY Hu Y Liang WH Ou CQ He JX. Clinical characteristics of coronavirus disease 2019 in China. medicine Engl J Med. (2020) 382:1708–20. 10.1056/NEJMoa2002032 PMC7092819 32109013

[23] Wang D Hu B Hu C Zhu F Liu X Zhang J. Clinical characteristics of 138 hospitalized patients with 2019 novel coronavirus-infected pneumonia in Wuhan, China. JAMA. (2020) 323:1061–9. 10.1001/jama.2020.1585 32031570 PMC7042881

[24] Guo T Fan Y Chen M Wu X Zhang L He T. Cardiovascular implications of fatal outcomes of patients with coronavirus disease 2019 (COVID-19). JAMA Cardiol. (2020) 5:811–8. 10.1001/jamacardio.2020.1017 32219356 PMC7101506

[25] Shi S Qin M Cai Y Liu T Shen B Yang F. Characteristics and clinical significance of myocardial injury in patients with severe coronavirus disease 2019. Eur Heart J. (2020) 41:2070–9. 10.1093/eurheartj/ehaa408 32391877 PMC7239100

[26] Chen N Zhou M Dong X Qu J Gong F Han Y. Epidemiological and clinical characteristics of 99 cases of 2019 novel coronavirus pneumonia in Wuhan, China: a descriptive study. Lancet (London, England). (2020) 395:507–13. 10.1016/S0140-6736(20)30211-7 32007143 PMC7135076

[27] Dmytriw AA Phan K Schirmer C Settecase F Heran MKS Efendizade A. Ischaemic stroke associated with COVID-19 and racial outcome disparity in North America. J Neurol Neurosurg Psychiatry. (2020) 91:1362–4. 10.1136/jnnp-2020-324653 32801118

[28] Song Z Xu Y Bao L Zhang L Yu P Qu Y. From SARS to MERS, thrusting coronaviruses into the spotlight. Viruses. (2019) 11:59. 10.3390/v11010059 30646565 PMC6357155

[29] Varga Z Flammer AJ Steiger P Haberecker M Andermatt R Zinkernagel AS. Endothelial cell infection and endotheliitis in COVID-19. Lancet (London, England). (2020) 395:1417–8. 10.1016/S0140-6736(20)30937-5 32325026 PMC7172722

[30] Mangalmurti N Hunter CA. Cytokine storms: understanding COVID-19. Immunity. (2020) 53:19–25. 10.1016/j.immuni.2020.06.017 32610079 PMC7321048

[31] Kihira S Schefflein J Mahmoudi K Rigney B B ND Mocco J. Association of coronavirus disease (COVID-19) with large vessel occlusion strokes: a case-control study. AJR Am J Roentgenol. (2021) 216:150–6. 10.2214/AJR.20.23847 32755225

[32] Carod-Artal FJ. Neurological complications of coronavirus and COVID-19. Rev Neurol. (2020) 70:311–22. 10.33588/rn.7009.2020179 32329044

[33] Valderrama EV Humbert K Lord A Frontera J Yaghi S. Severe acute respiratory syndrome coronavirus 2 infection and ischemic stroke. Stroke. (2020) 51:e124–e7. 10.1161/STROKEAHA.120.030153 32396456

